# The Non-Invasive Prediction of Colorectal Neoplasia (NIPCON) Study 1995–2022: A Comparison of Guaiac-Based Fecal Occult Blood Test (FOBT) and an Anti-Adenoma Antibody, Adnab-9

**DOI:** 10.3390/ijms242417257

**Published:** 2023-12-08

**Authors:** Martin Tobi, Fadi Antaki, Mary Ann Rambus, Yu-Xiao Yang, David Kaplan, Rebecca Rodriguez, Benedict Maliakkal, Adhip Majumdar, Ereny Demian, Yosef Y. Tobi, Paula Sochacki, Murray Ehrinpreis, Michael G. Lawson, Benita McVicker

**Affiliations:** 1 Department of Research and Development, John D. Dingell VAMC, Detroit, MI 48201, USA; fadi.antaki@va.gov (F.A.);; 2 Department of Research and Development, Philadelphia VAMC, Philadelphia, PA 19104, USAdakaplan@mail.med.upenn.edu (D.K.); rmrodriguez126@gmail.com (R.R.); 3 Nashville Hospital Association, Nashville, TN 37027, USA; 4 Departments of Medicine, State University of Pennsylvania, State College, PA 16802, USA; edemian@pennstatehealth.psu.edu; 5 New York Medical College, Touro University, Valhalla, NY 10595, USA; 6 Detroit Medical Center, Wayne State University School of Medicine, Detroit, MI 48201, USA; 7 Kaiser Permanente Research Foundation, Sacramento, CA 95825, USA; 8 Omaha VAMC, Omaha, NE 68105, USA

**Keywords:** fecal occult blood, adenoma, Adnab-9, p87, stool, urine, saliva

## Abstract

Given the need to improve the sensitivity of non-invasive methods to detect colorectal neoplasia, particularly adenomas, we compared a fecal test using a monoclonal antibody (Mab) raised against constituents of colonic adenomas designated Adnab-9 (Adenoma Antibody 9), recognizing an N-linked 87 kDa glycoprotein, to gFOBT, which is shown to reduce CRC mortality. p87 immunohistochemistry testing is significantly more sensitive (OR 3.64[CI 2.37–5.58]) than gFOBT (guaiac-based fecal occult blood test) for adenomas (<3 in number), advanced adenomas (OR 4.21[CI 2.47–7.15]), or a combination of the two (OR 3.35[CI 2.47–4.53]). p87 immunohistochemistry shows regional Paneth cell (PC) expression mainly in the right-sided colon and is significantly reduced in the ceca of African Americans (*p* < 0.0001). In a subset of patients, we obtained other body fluids such as urine, colonic effluent, and saliva. Urine tests (organ-specific neoantigen) showed a significant difference for advanced adenomas (*p* < 0.047). We conclude that fecal p87 testing is more sensitive than gFOBT and Adnab-9 and could be used to better direct the colonoscopy screening effort.

## 1. Introduction

In 2020, one-third of 147,000 colorectal cancer (CRC) patients diagnosed in the US died of their disease [1]. Currently, the guaiac-based fecal occult blood test (gFOBT) is the only screening modality that has prospectively reduced the incidence and mortality from CRC [2]. More effective screening strategies are needed to eliminate CRC precursors, adenomatous and serrated polyps [3,4,5]. While there have been advances in non-invasive CRC testing which largely relies on FIT components, they are relatively expensive with a modest sensitivity for the adenomatous polyp precursors [6].

Our previous retrospective data suggest sensitivity of Adnab-9 binding in CRC of about 60% in both stool and effluent colonic washings [7,8,9]. We demonstrate a colonic p87 field effect (FE) of carcinogenesis [10] confirmed via immunohistochemistry herein. The convenience of urine testing was initially explored in patients with CRC using the BAC18.1 monoclonal antibody that recognizes urinary organ-specific neoantigen (OSN). OSN was highly correlated with CRC in a past blinded study [11] and herein the utility for adenoma detection is explored, as was recently investigated in [12].

## 2. Results

**Figure 1:**
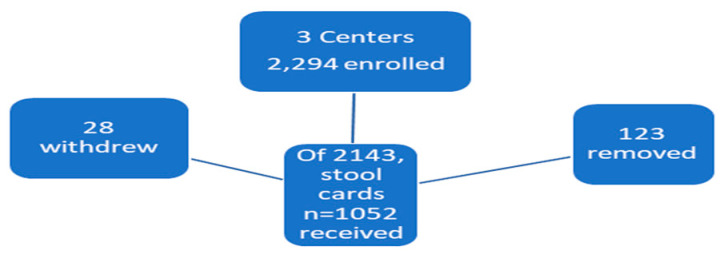
Enumerated Schematic Study Outline.

Figure 1 shows the participant accumulation and attrition due to withdrawal and removal from the study. Reasons for withdrawal were complaints of old age, preference to see non-VA physicians, time restrictions, the feeling that they had already participated in the study, and a sense of comorbidity.

Participant removals were likewise varied and usually related to non-eligibility, non-compliance with mailing samples, failure to present for colonoscopy, and issues of non-compliance with completing the necessary questionnaires.

Initially, 2294 participants signed consents and 1052 mailed in stool tests. This result represents a response of 46%, which is similar to the response for non-study fecal occult blood returns at the Detroit VAMC (Dr. Alan Pawlow personal communication). There were 2169 respondents from Detroit, 67 from Albany VAMC, and 58 from the Philadelphia VAMC. The vast majority were from the Detroit VAMC, which represented 94.6% of the entire sample. Demographic data are provided in Table 1.

Table 2 tabulates the various study parameters for the largest site only for the Detroit patients where long-term follow-up was available.

**Figure 2:**
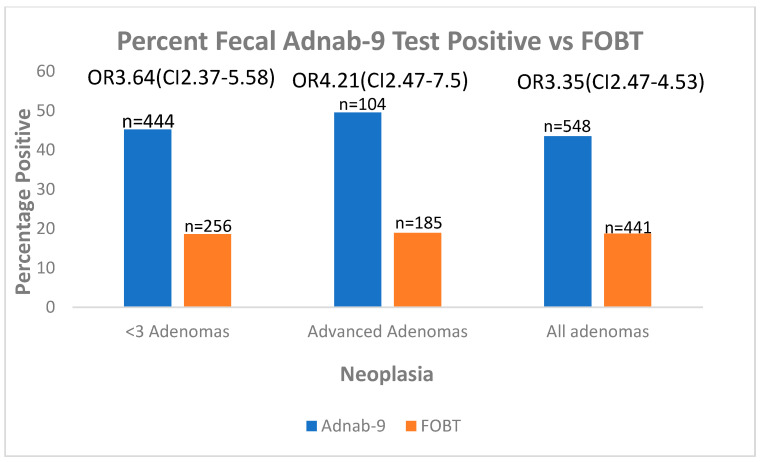
Shows the results for fecal Adnab-9 testing compared with that of gFOBT for adenomas, advanced adenomas, and combined adenomas.

Figure 2 shows the proportion of fecal Adnab-9 positive testing is significantly higher than that of gFOBT for all categories. There was no designated average risk control group. For phase 2 patients with additional studies performed, rectal colonic washings were aspirated on intubation, and effluent was assayed for protein as with stool and used as the standard for ELISA. Values of optical density > 0.05 or 2 standard deviations above background were regarded as positive.

**Figure 3:**
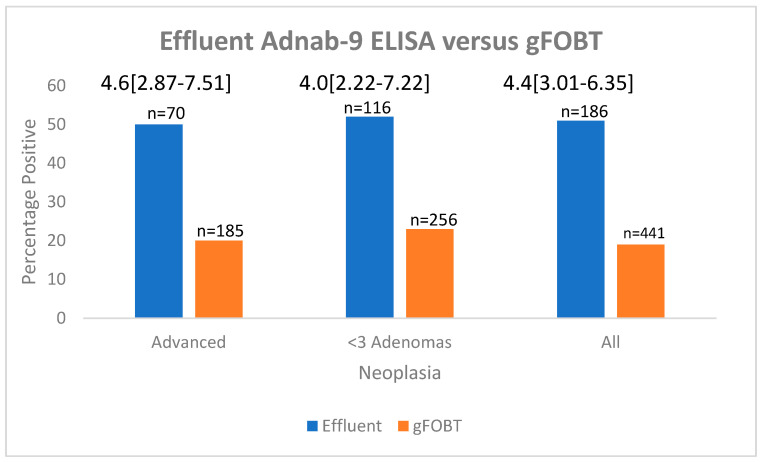
Bar diagram with percentages of effluent samples compared with gFOBT in patients with adenomas.

Figure 3 is a bar graph that shows that Adnab-9 testing has a greater proportion of Adnab-9 positivity as opposed to gFOBT-positive results.

Urinary OSN was assayed in 107 urine samples and analysis was conducted using Student’s *t*-test in the following three groups: no neoplasia; small adenomas up to two in number; and advanced adenomas (1 cm or greater in size at index colonoscopy; villous features or three or more in number). Patients with advanced adenomas had greater mean OSN values than patients with no or small adenomas (Table 3).

### Phase 2 Studies

Saliva was collected in 31 patients (10 AAs and 21 Caucasians), 21 were males and 10 were females; the mean age ± standard deviation was 57.9 ± 11.6 years with no polyps in 9 patients of group 0, 11 in group 1, and 10 in group 2. Adnab-9 saliva ELISA was standardized using protein content as with the fecal test and the cutoff was similar. There were no significant differences between the means (0.019 ± 0.016; 0.013 ± 0.011; and 0.015 ± 0.023, respectively). There was a mild trend between the first 2 groups (*p* = 0.22).

**Figure 4:**
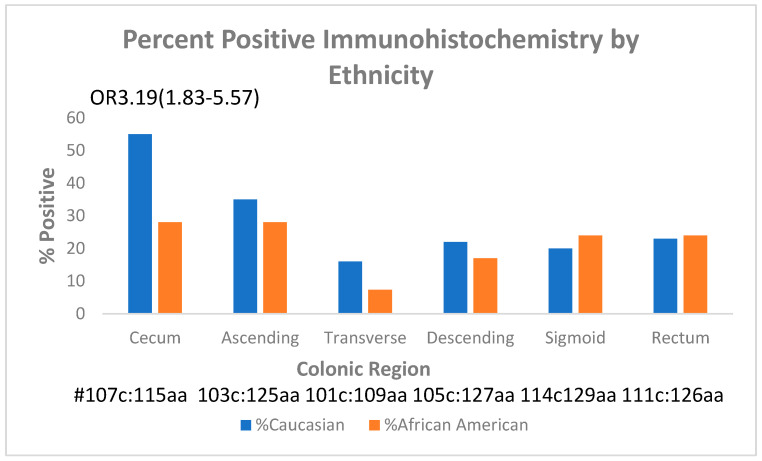
Bar diagram of p87 immunohistochemistry: percent labeling in the regions of the colon in AA and Caucasian patients.

Regarding the distribution of p87 in the colon, IHC revealed that cecum and ascending colon had the highest labeling with a significant decrease in the cecum between Caucasian and AA patients (Figure 4).

In the fixed IHC, Caucasian (c) ceca scored significantly higher than AA (aa). With native antigen in extracted tissue, the only significant differences were in the recta, where AA scored significantly lower (*p* < 0.03). Numbers (#) are provided for comparison.

The significance of native antigen expression, significant only in the rectum, is currently unclear but would appear to be the mirror image of the fixed antigen in the cecum. The major question was whether there was a difference in the expression in the field effect (FE) of p87 and whether this was relevant to neoplasia. This does not appear to apply when considering all patients; however, when comparing insignificant to significant neoplasia, the presence of neoplasia in a colon that has no FE was 61.4% as opposed to the existence of any FE, in which the percentage was 40.5%, which appears to be protective (OR0.43[CI 0.19–0.97]; *p* < 0.045).

**Figure 5:**
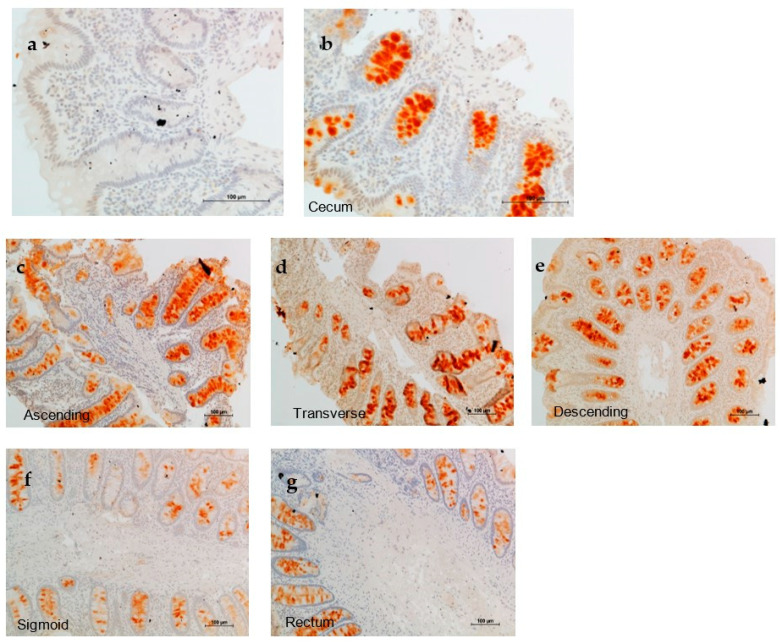
(**a**–**f**) Show a representative gradient of labeling in the colon with maximal intensity in the cecum and least intensity in the rectosigmoid. Adnab-9 labeling in the cecum (negative control (**a**) and Adnab-9 antibody stained (**b**), magnification 20×). Low-power photomicrographs of p87 staining from the ascending colon to the rectum (**c**–**g**). Magnification 10×. The counterstain was hematoxylin.

The significant inverse correlation between regional Figure 5 scores and the proportional percentage of the entire group suggests a relationship but only a mild trend when comparing regional means to Figure 5 scores (related data are displayed in Table 4). The comparison does not exclude an observation that there is a relationship that suggests that the selected Figure 5 set was an appropriate example of staining, particularly on the left side of the colon.

Prediction of future neoplasia based on p87 stool tests was also of interest and future adenoma development was followed. It was found that 58% of 361 patients with future colonoscopies were accurately predicted. Accuracy was significantly higher (OR1.55[1.01–2.38]; *p* = 0.051) in 202 AAs (62.4%) versus 153 Caucasians (51.6%).

**Figure 6:**
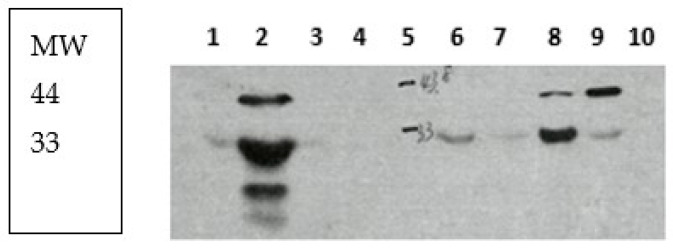
Western blot of stool extracts.

Figure 6 shows representative distinct bands in lane 2 (graded as 3), test stools in lanes 1, 3, 4, 6, and 7 (very faint 33 kDa bands—graded as 0.5), and 8 and 9 (clearly discernable (graded as 1) 44 and 33 kDa bands—graded as 2), with lane 10 devoid of bands—graded as 0. Lane 5 shows molecular weight band markers. The box at right shows molecular weight (MW) markers. Clinical details are shown in Table 5.

We compared the strength of bands as graded above with the highest OD values of stool or effluent, which yielded a statistically significant positive correlation analysis (r coefficient of 0.716 and *p*-value of *p* < 0.031). This suggests a relationship between outcomes of p87 tests for stool and supports Adnab-9 antibody specificity and the importance of protein standardization for these tests.

## 3. Discussion

The NIPCON study spanned 27 years and the primary goal was to compare adenoma detection using gFOBT and Adnab-9 MAb. Adnab-9 was found to be more sensitive than gFOBT in both small and advanced adenomas in both stool and testing of colonic effluent. While gFOBT+ results invited intervention in some cases, indications varied widely. While newer tests can equal the sensitivity of Adnab-9 for neoplasia, its sensitivity for non-advanced adenomas is likely superior [6] by half (50 vs. 25%), although a head-to-head comparison has not been performed.

We performed detailed studies for FE expression via IHC to try to elucidate p87 antigen origins. We found a colonic trend, with the cecum predominating and decreasing distally. This mirrors the distribution of PC in the colon, which appears to be part of the GI tract (GIT) innate immune system (InImS). Despite significantly less cecal expression in the cecum of AA patients compared with Caucasians, there was not a commensurate change in CRN in these primary groups, although it appears that an FE [10] might protect against CRN. An independent study showed a survival advantage with Adnab-9 staining in early-stage CRC (please see Table 5 in Reference [14]). Of interest is the similarity of this prospective study’s sensitivity with the retrospective data from a study performed in China [4], where the incidence of CRC was about one-third of that of the US.

While we were successful using urine with OSN testing, this was only significant with advanced adenomas. We have previously published [11] successful detection of CRC using this form of testing; moreover, urinary testing may be more acceptable to the general population than stool collection. We were not successful using unstimulated saliva but the numbers were rather limited.

The recently published NordICC study [15] supports our contention with respect to colonoscopy for which there was no survival advantage. In addition, from the same group, a meta-analysis of 2.1 million people showed no survival advantage of commonly practiced screening tests including CRC [16]. In that study, only flexible sigmoidoscopy may have possibly conferred an additional 110 days of life with no advantage of guaiac fecal occult blood (gFOBT) testing (amongst others). Their first study [15] was criticized for a low adenoma detection rate [17]. However, we attempted to deflect that criticism [18] as the European population in question was healthier than that of the United States used as a basis [19] for ADR comparison. In this study, we show that p87 fecal testing for adenomas is superior to gFOBT, allowing for the selection of a population at higher risk for pre-malignant polyps for targeted colonoscopic screening of CRC adenoma precursors. We have long contended that colonoscopy was adopted as a screening tool without the requisite scientific rigor and termed the rush to implement it as the “Cecal Stampede” [20].

Of interest is the increased CRN prevalence in AAs with low vitamin D levels. While this has recently been reported for colorectal cancer [21,22], we show that this also applies to adenomas; this supports the hypothesis of the authors of that article, which was drawn from a female database. In our study with an AA male predominance, we were able to show significantly more adenomas in males with low vitamin D levels than those with normal levels. The results were similarly significant in Caucasian versus AA males, *p* < 0.049.

Our study was a cross-sectional, prospective study focusing on the detection of adenomas and there are few other studies that placed a priority on this. An overview of screening methods was recently published [23] and placed an emphasis, amongst others, on maximizing screening sensitivity and cost-effectiveness. They advocate putting in place public health policies alongside effective biomarkers to solve screening problems and quote others that suggest a 90% reduction in mortality is tenable [24]. In a case in point, they criticize the currently available multi-target stool DNA tests for a sensitivity of 42.4% and conclude that even colonoscopy is labor-intensive and invasive and suggest instead to develop new fecal and gut bacterial biomarkers. Others agree with the latter approach and voice doubt regarding the validity of detecting adenomas as part of the screening effort [25] and decry the excessive cost of $600 for multitargeting fecal testing which may defeat the goal of screening cost-effectiveness. Once the stool biomarker test returns as positive, the colonoscopy must be sensitive enough to decrease the economic cancer burden [26] by detecting and removing adenomas, but the standard adenoma detection rate (ADR) is highly variable (7–53%). To overcome this problem, some have advocated for the identification of polyps using deep learning convolutional neural networks (CNNs) to augment ADR [27]. They report that CNN missed 12% of 678 polypoid polyps and 11% of non-polypoid adenomas, which is not much higher than the missed cancer rate in the US and certainly much higher (17%) for missed adenoma rates reported by Korean authors [28]. However, of the eight versions available, none could work in real time but one working on sub-patches of images did achieve an accuracy of 91% for polyp detection [29]. Most of these studies are case–control or retrospective based on stool biorepositories. A fresh approach championed by Chinese researchers systematically probed the patient microbiome based on specific microbes closely associated with colorectal neoplasia including adenomas using metagenomic analysis of a fecal *Lachnoclostridium* species designated m3 [30]. While not surpassing prospective p87 sensitivity adenoma detection, they did achieve retrospective 51% sensitivity using m3, to arrive at a parity. While this may bode well for future combinations of m3 with p87 or fecal immune testing (FIT), the current understanding of microbiome detection biomarkers for colorectal adenomas is limited despite many recent advances in this crowded, but highly specific field [31,32,33,34,35,36,37,38,39,40].

The major pitfall of our study was the lack of an average-risk cohort that might be more relevant to colorectal cancer screening. We lack a cost analysis although the likely cost would approximate FIT testing. Since this was largely a VHA study, the results are largely applicable to males, although we made the effort to recruit more females and succeeded in doubling the expected participation. While we invoke the GIT InImS, this is based on the fact that p87 is a PC marker [41,42,43], but the actual functionality requires further research.

## 4. Materials and Methods

### 4.1. Participants

VHA centers in Detroit, Albany, and Philadelphia recruited participants scheduled for colonoscopy by their primary care providers. A short screening questionnaire for inclusion and exclusion identified an asymptomatic, high-risk cohort by the affirmation of a history of personal colorectal neoplasia (CRN), past or present inflammatory disease, family history of CRN, unexplained anemia, a family history of CRN, or history of abdominal symptoms. Patients with a bleeding disorder or those unwilling to be a participant were excluded from the study. Following informed consent, patients were issued four Hemoccult II (mostly SmithKline Diagnostics, San Jose, CA, USA) guaiac (gFOBT), with instructions for dated sequential daily collection, and mailing using a pre-stamped, insulated envelope. A urine specimen was requested at the time of consent or before index colonoscopy and an aliquot was sent to the clinical laboratory for creatinine estimation. The patient’s personal information was entered into a secured database including demographics and relevant parameters such as iron studies, BMI, and other biological data.

These patients filled in a more detailed questionnaire regarding medications taken, and other maladies, and signed a separate phase 2 consent. About 10% of patients were offered participation in a phase 2 study where 4 forceps biopsies were obtained, 3 from the cecum, ascending, transverse, descending, sigmoid, and rectum for immunohistochemistry and the fourth for extraction, using Adnab-9. Saliva was similarly processed. Repeat sampling on colonoscopy follow-up was initially limited to 3 years but extended to 5 years by the reviewers, upon competitive renewal. The grading of findings on the index colonoscopy was as follows: 0—no neoplasia; 1—2 or less small (<1 cm) adenomas; 2—>2 adenomas or >1 cm; or high-grade dysplasia. CRC was not included. From a historical perspective of evolving polyp nomenclature, the study was initiated within 5 years of the reported proposal of serrated lesions in 1990 [43,44] but the proposed nomenclature was not accepted until 2003 [45], 8 years after the study was initiated. Thus, only later participants had this diagnosis universally accepted, which was regarded as the equivalent of advanced lesions, and designated as grade 2.

### 4.2. Adnab-9 Stool ELISA

p87 expression measured via Adnab-9 ELISA was performed as previously described [7,8,9]. In brief, windows were excised from hemoccult cards from multiple manufacturers; thus, the make of the card might have varied. Extracted stool from the cutout was vortexed in chilled buffered saline, centrifuged at 10,000× *g* for 10 min, and supernatants were stored at 80 °C for protein determination. Unstimulated saliva supernatant and voided urine samples were assessed for protein content (saliva) and urine creatinine content was determined and placed in frozen storage.

### 4.3. ELISA and Western Blotting

Protein content was determined using the Bradford or Lowry methods [8,9] and dilutions of each stool yielding 5 µg of protein/well of a 96-well plastic microtiter plate were loaded in triplicate (Nunc, Copenhagen, Denmark). Wells were blocked with a 5% bovine serum albumin (Sigma, St. Louis, MO, USA) to prevent non-specific binding. One set of wells was incubated with a 1:500 dilution of Adnab-9 and the control set with an irrelevant mouse monoclonal antibody (Mab) as a negative control, adenoma extract as the positive control, and background estimated. Values of OD > 0.05 or 2 SD > background were considered positive once plates were processed according to the manufacturers’ specifications and OD was measured (Titertek Multiscan, Flowlabs, McClean, VA, USA). For the OSN urine assay, wells were standardized with 200 µg of creatinine and the primary BAC18.1 antibody (gifted by Prof. Zvi Bentwich); OD-background >1.5 was considered positive.

For extract Western blotting, homogenized biopsy or fecal samples were centrifuged (5000 rpm) and the supernatant was subjected to a 5 min ultrasonication. Then, after a final 10,000 rpm centrifugation, the protein content was determined. An amount of 10 µg of extracted sample was loaded onto lanes of a 9.6% SDS-polyacrylamide gel (Sigma) and electrophoresis was carried out followed by transfer to a polyvinylidene difluoride membrane (Kirkegaard & Perry, Gaithersburg, Maryland, USA). Membranes were reacted with Adnab-9 and binding was detected using an ABC test kit (Vector Laboratories, Newark, CA, USA).

### 4.4. Immunostaining

Immunohistochemistry (IHC) was performed using the aforementioned kit (Vector Laboratories, Newark, CA, USA) to stain 5 µm thick tissue paraffin sections after deparaffinization, which were then stained with 1:500 dilution of Adnab-9 (available at the time from Dako Inc., Carpinteria, CA, USA), with the control slides stained with an irrelevant monoclonal, using the same avidin-biotin complex (ABC) method as above. The grading of results was conducted as previously reported [41].

Briefly, following 30 min of blocking in 2.5% normal horse serum, the sections were incubated overnight at 4 °C with Adnab-9 antibody and control sera. After washings, the sections were incubated with goat anti-mouse IgG antibody followed by treatment with polymer antibody and detection with ImmPACT DAB reagent (Vector Labs). Images were captured for at least five random fields per section using a Nikon Eclipse 80i microscope and DS-Fi2 camera (Nikon Inc., Konan, Minato-ku, Tokyo, Japan).

### 4.5. Statistics

We utilized online statistics available as the Vassarstats URL (http://vassarstats.net/, accessed on 5 December 2023.) and both ordinal and non-ordinate data were processed, including regression correlations. Proportional data were evaluated for significance via chi-square and statistical difference of means via Student’s t-test. The normality of values was tested using the online Kolmogorov–Smirnov calculator https://www.socscistatistics.com/tests/kolmogorov/, accessed on 5 December 2023. Mann–Whitney nonparametric testing was used depending on the normality distribution. The *p*-values were considered significant at the <0.05 level, with a power of 80% for the detection of a β value of 0.2 with a two-sided value of α 0.01.

## 5. Conclusions

This study is one of the longest colorectal cancer studies focused on polyp detection. While there have been longer surveillance studies [46] and even longer polyp studies [47], the latter also lacked a control group and relied on retrospective SEER data. Our study was entirely prospective and multifaceted with a multiplicity of endpoints. Our veteran partners were involved at the outset and signed petitions were obtained from members of most service organizations calling for a study of this scope and nature. Our partners are our cheerleaders; therefore, without their support, this study would not have been initiated. Whatever we have achieved, they are equal partners and we salute them. We also come away from this study with a greater understanding of the utility of various bodily fluids and colonic distribution of expression of a Paneth cell marker and demographic implications. We share setbacks with predecessor studies such as not having a control group but avoided using external retrospective databases to retain our prospective study attributes. The aforementioned studies definitely helped us to guide the construction of our study tenets and we are most grateful to their authors for their guidance and direction. Naturally, as technology advances, other biomarkers will come to the fore and we mention some for our readers’ interest in References [48,49]. In a relatively recent comprehensive review [50] of IHC staining methods, Adnab-9 was one of 8 other markers selected (p53, β-catenin, COX2, Ki-67, Cyclin D1, Annexin A10, Aldehyde Dehydrogenase Isoform 1A1). 

## Figures and Tables

**Table 1:** Patient demographics for the three study sites.

Parameter (Total)	Detroit	Albany	Philadelphia
Participants (2294)	2169	67	58
Withdrew (28)	27	1	0
Removed (123)	115	8	0
Sex Males:Females (unk)	1929:240	9:(58)	41:12(5)
Age x ± sd	60.54 ± 12.32	64.45 ± 9.35	54.68 ± 12.49
Ethnicity His/AA/C (unk)	28/1082/1026 (33)	C34:(33)	4/16/18(20)

Footnotes: Unk—unknown; x—mean; sd—standard deviation; His—Hispanic; AA—African American; and C—Caucasian.

**Table 2:** Risk factors and follow-up of Detroit patients for the life of the study or until death.

Parameter	Years (Proportions)	African American *n* = 1101	Caucasians *n* = 1114
Mean follow-up live Pts	14.56 ± 7.99 med 15.9	14.45 ± 5.33	14.29 ± 5.16
Mean f/u deceased Pts stool	9.25 ± 9.07 med 8.58	10.12 ± 11.45	8.36 ± 5.08
Survival alive/dead	1140/909 55.6% alive	577/472 (55.01%)	512/412 (55.4%)
Age at enrollment (years)	60.52 ± 12.31 med 60	60.27 ± 12.7	60.79 ± 12.45
Smoking status active/never/quit	554/374/230 Active: 47.84%	330/182/113 (52.8%). 1.46(1.16–1.85); *p* < 0.002.	224/179/114 (43.33%)
Chronic hepatitis C	178 of 931- 19.1%	133 of 533: 24.95% 2.86(1.94–4.20); *p* < 0.0001	39 of 374: 10.43%
Family history cancers	828 of 2682- 30.9%	400 of 900 (44.44%) 3.67(1.09–1.66); *p* < 0.0001	385 of 1000 (38.5%)
Personal history cancer	228 of 1854: 12.3%	180 of 956 (18.83%) 1.69(2.95–4.57); *p* < 0.0001	105 of 968 (12.10%)
Compliance DCBE	317 of 1987 (15.95%)	190 of 1021 (18.61%) 1.47(1.15–1.88); *p* < 0.003	121 of 898 (13.47%)
Metformin	80 of 390 (20.51%)	35 of 218 (16.06%)	44 of 161 (27.33%) 1.97(1.19–3.25): *p* < 0.011
Illicit drug use	102 of 632 (16.13%)	77+ 289- (21.0%) 2.61(1.59–4.29); *p* < 0.0002.	23 of 248 (9.27%)
Vitamin D blood level ng/mL mean ± standard deviation	23.00 ± 12.03 *n* = 358	21.82 ± 12.16 *n* = 209 *p* = 0.4 AA versus Caucasian.	25.07 ± 10.85 *n* = 149
Significant proportional differences	N/A	Males deficient with concentrations < 30 ng/mL: 51.3% AA vs. 25.85% Caucasian OR3.16(1.25–8.01); *p* < 0.02.	Males deficient in 25.85%; <30 51% deficient
Female vs. Male survival 55–64-year age group *	OR3.14(1.98–5.00) *p* < 0.0001	OR2.71(1.74–4.20); *p* < 0.0001	OR3.07(1.91–4.93) *p* < 0.0001

Footnotes::https://www.statista.com/statistics/241572/death-rate-by-age-and-sex-in-the-us/ 18 October 2023 [13]; med-median; females * outlived males by 66.7% OR1.67(1.51–1.84) *p* < 0.0001. There were no significant differences between alcohol intake, diabetes, BMI, and F/H CRC; FOBT and flexible sigmoidoscopy or colonoscopy compliance; NSAID and statin use; history of/or cumulative adenomas; normal colonoscopy, cancers, and insignificant or advanced adenomas at the index colonoscopy; qualitative or quantitative fecal Adnab-9 or Western blot; Adnab-9 FE in stool or effluent; % females; and % male or female survival/age. The reader is directed to note that most of the above attributes are correlated with adenoma tumorigenesis. DCBE-Double contrast barium enema.

**Table 3:** Comparison of urinary OSN ELISA results in three groups of patients.

Group	Number	Mean Values	Standard Deviation	*p*-Values
No neoplasia	59	1.724	1.080	Basis of comparison
Minor adenomas	25	1.712	1.080	0.96
Advanced adenomas	23	2.274	1.162	<0.047

OSN means between AAs and Caucasians in each group were similar (*p* = 0.91; *p* = 0.56; and *p* = 0.28, respectively). AAs were well represented in all 3 groups (49, 65, and 59 percent, respectively). Women were better represented in the no neoplasia group (18.6%) versus the adenoma (8%) and the advanced adenoma group (4.4%) but not statistically significantly so (*p* = 0.084). Of interest, there was a mild trend toward agreement in the advanced adenoma group between outcomes of urinary OSN and fecal Adnab-9 testing in the groups (45, 42, and 67%, respectively; *p* = 0.11).

**Table 4:** A comparison of the regional mean of scores of IHC with scores depicted in Figure 5.

Region	Cecum	Ascending	Transverse	Descending	Sigmoid	Rectum
Number	195	163	209	209	213	226
% Stained	72	87	92	92	94	91
Figure 5 #: % All	5+: 1.3	3+: 4.3	4+: 0	2+: 13.6	0.5+: 53	1+: 29
IHC x ± sd	0.419 ± 0.665	0.347 ± 0.621	0.210 ± 0.474	0.199 ± 0.468	0.230 ± 0.522	0.254 ± 0.557

Footnotes to Table 4: #—Score number of Figure 5 below; x—mean; and sd—standard deviation via semiquantitative grading. There was a significant inverse correlation of % of all versus Figure 5 regional scores, r = −0.9; *p* < 0.021. There was a positive correlation mild trend of Figure 5 scores and the mean regional scores of the regional groups, r = 0.6; *p* = 0.22.

**Table 5:** Patient characteristics.

Lane #	Age Gender Ethnicity	Colorectal Neoplasia	Band Strength, ELISA OD Smoker
1	53 yr male, Caucasian	Small adenoma of TC	±33 kDa, OD 0.030 45 pyh
2	79 yr male, Caucasian	Sigmoid adenoma	Strong, multiple ODE 0.659 65 pyh *
3	55 yr male, African Am	Small cecal/TC adenomas	±33 kDa OD 0.100+ ODE 0.007 60 pyh *
4	77 yr male African Am	Small sigmoid adenomas	±33 kDa OD 0.007 ODE 0.040 60 pyh *
6	51 yr male African Am	Ulcerative colitis	+33 kDa OD 0.034 ODE 0.000 15 pyh #
7	51 yr male Caucasian	F/H GI cancers (PC Gf)	±33 kDa OD 0.117 20 pyh
8	61 yr female Caucasian	Two small DC adenomas	+44/30 kDa OD 0.100 ODE 0.005 @^Quit
9	64 yr male African Am	Small polyp TC, F/H CRC Pa	+44/33 OD 0.024 ODE 1.060 #^; Quit
10	38 yr male Hispanic	Poor prep F/H Ma Fem gen	No bands OD 0.001 (low stool protein)

Footnotes: Am—American; TC—transverse colon; DC—descending colon; yr—years old; kDa—molecular weight of band in kilodaltons; pyh—pack year history of smoking; *—lung cancer; #—large cell lymphoma mass in liver; @—breast cancer; ^—acute myeloid leukemia; #^—marginal zone lymphoma; OD—optical density; ODE—effluent OD; F/H—family history; Pa—father; Ma—mother; and Gf—grandfather. Fem gen-female genital tumor.

## Data Availability

Data embodied in the manuscript.
